# Single Molecule FRET Analysis of CRISPR Cas9 Single Guide RNA Folding Dynamics

**DOI:** 10.1021/acs.jpcb.2c05428

**Published:** 2022-12-23

**Authors:** Ikenna C. Okafor, Taekjip Ha

**Affiliations:** † Department of Biology, Johns Hopkins University, Baltimore, Maryland 21218, United States; ‡ Department of Biophysics, Johns Hopkins University, Baltimore, Maryland 21218, United States; § Department of Biophysics and Biophysical Chemistry, Johns Hopkins University School of Medicine, Baltimore, Maryland 21205, United States; ∥ Department of Biomedical Engineering, Johns Hopkins University, Baltimore, Maryland 21218, United States; ⊥ Howard Hughes Medical Institute, Baltimore, Maryland 21205, United States

## Abstract

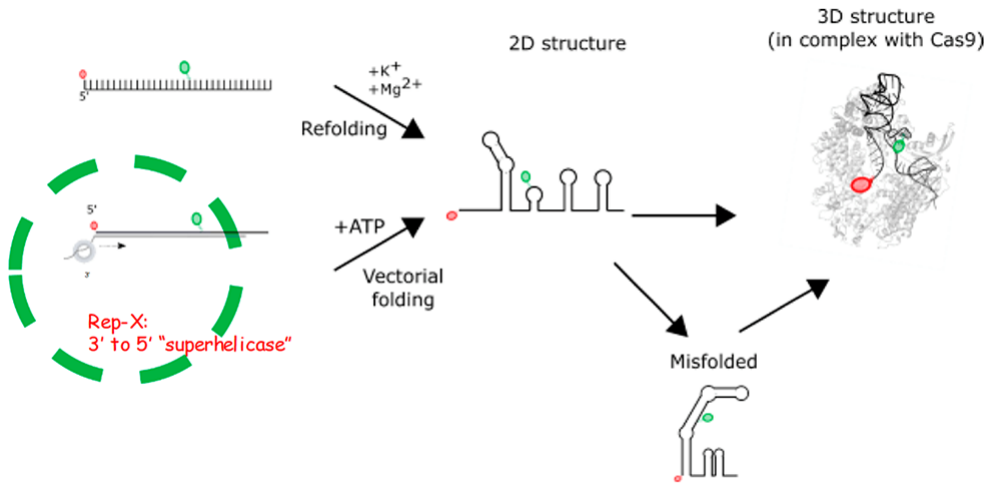

CRISPR Cas9 is an RNA guided endonuclease that is part of a bacterial adaptive immune system. Single guide RNA (sgRNA) can be designed to target genomic DNA, making Cas9 a programmable DNA binding/cutting enzyme and allowing applications such as epigenome editing, controlling transcription, and targeted DNA insertion. Some of the main hurdles against an even wider adoption are off-target effects and variability in Cas9 editing outcomes. Most studies that aim to understand the mechanisms that underlie these two areas have focused on Cas9 DNA binding, DNA unwinding, and target cleavage. The assembly of Cas9 RNA ribonucleoprotein complex (RNP) precedes all these steps and includes sgRNA folding and Cas9 binding to sgRNA. We know from the crystal structure of the Cas9 RNP what the final sgRNA conformation is. However, the assembly dynamics has not been studied in detail and a better understanding of RNP assembly could lead to better-designed sgRNAs and better editing outcomes. To study this process, we developed a single molecule FRET assay to monitor the conformation of the sgRNA and the binding of Cas9 to sgRNA. We labeled the sgRNA with a donor fluorophore and an acceptor fluorophore such that when the sgRNA folds, there are changes in FRET efficiency. We measured sgRNA folding dynamics under different ion conditions, under various methods of folding (refolding vs vectorial), and with or without Cas9. sgRNA that closely mimics the sgRNA construct used for high resolution structural analysis of the Cas9-gRNA complex showed two main FRET states without Cas9, and Cas9 addition shifted the distribution toward the higher FRET state attributed to the properly assembled complex. Even in the absence of Cas9, folding the sgRNA vectorially using a superhelicase-dependent release of the sgRNA in the direction of transcription resulted in almost exclusively high FRET state. An addition of Cas9 during vectorial folding greatly reduced a slow-folding fraction. Our studies shed light on the heterogeneous folding dynamics of sgRNA and the impact of co-transcriptional folding and Cas9 binding in sgRNA folding. Further studies of sequence dependence may inform rational design of sgRNAs for optimal function.

## Introduction

CRISPR is an immune system in bacteria and archaea that allows them to defend themselves against phages and viruses. The two main steps for carrying out this process are immunization and interference. A piece of the foreign genetic sequence is stored at the CRISPR locus during the immunization step. Upon secondary exposure, the sequence is transcribed into a noncoding RNA. The RNA guides CRISPR nucleases to foreign nucleic acid to initiate degradation in the interference step.^1^ The many types of CRISPR systems differ in the way that they carry out these steps. In the most widely studied type-II A systems, the interference step requires the Cas9 RNA guided endonuclease, the CRISPR RNA (crRNA), and the transactivating crRNA (tracrRNA). The crRNA and tracrRNA bind each other through sequence complementarity to form the guide RNA (gRNA). Cas9 and the gRNA assemble into a ribonucleoprotein (RNP) complex, which binds and cuts the invading viral or phage DNA.^2−4^

This system has been adapted for genome editing applications in eukaryotic cells. For these applications, a single gRNA (sgRNA), a crRNA and tracrRNA chimera, is most commonly used. Cas9 can be targeted to almost any genomic sequence by rewriting the sgRNA. This makes Cas9 RNP a programmable gene-editing tool.^5,6^ While Cas9 RNP is widely used for research purposes, variability in editing outcomes is hindering an even wider adoption. Previous work has highlighted the importance of sgRNA structure and design in dictating the editing outcomes.^7−13^ So far, the sgRNA structure–function relationship has only been studied in the context of Cas9 DNA binding, unwinding, and cleavage.^7,10,11,14−16^ Although RNP assembly precedes these steps, studies directly investigating the mechanism of Cas9 RNP assembly are lacking. Previously, DNA binding or cleavage has been used as a readout for RNP assembly.^17,18^ However, these approaches lack the spatial and temporal resolution necessary to study the key details of this reaction such as sgRNA conformational dynamics. To address this, we developed a single molecule fluorescence resonance energy transfer (smFRET)^19^ assay to study sgRNA dynamics in the context of Cas9 RNP assembly.

Single-molecule measurements can reveal multiple subpopulations and interconversion between them in the most direct manner. For example, static and dynamic heterogeneities in single enzyme reactions were discovered by the laboratory of Sunney Xie more than 20 years ago.^20^ Relevant to this work, RNA folding, RNP assembly and dynamics, CRISPR Cas9 activities have been extensively studied using single-molecule fluorescence and mechanical manipulation tools.^21−35^ In many Cas9-based applications, sgRNA is transcribed in the cell, and potential misfolding during transcription could be detrimental. In some other applications, Cas9-sgRNA is delivered to the cell as a preassembled RNP. Here, we measured sgRNA folding by itself and when it is aided by Cas9. We also compared sgRNA refolding, where a fully synthesized RNA is allowed to fold by adding high salt solution, and vectorial folding, where co-transcriptional folding of sgRNA is mimicked by using a helicase-catalyzed unwinding of an RNA-DNA duplex to reveal the RNA in the direction of transcription and at the speed of transcription.^29,30^

## Experimental Methods

### Preparation of Dual Labeled sgRNA and Cas9

Two fragments, 59nt and 45nt, for sgRNAs were synthesized by Integrated DNA Technologies (IDT). The 59nt fragment contained a Cy5 fluorophore at the 5′ end. Cy3 *N*-hydroxysuccinimido (NHS) was attached to the 45 nt RNA through a thymine modified with an amine group through a C6 linker (IDT code: /iAMC6T/). Labeling efficiency for both fluorophores was higher than 50% as assessed using the NanoDrop 2000 spectrophotometer. We used splint ligation to link the two fragments together. The DNA splint was synthesized by IDT. Labeled RNA fragments were annealed to the DNA splint by heating the mixture to 95 °C in T50 (10 mM Tris-HCL, pH 8, 50 mM NaCl) and gradually cooling to 5 °C over 2 h. T4 RNA ligase 1 (NEB: M0204S) was used to ligate the two fragments. We separated the ligated sgRNA from fragments and the DNA splint by running the sample on a 12% urea denaturing poly acrylamide gel. The fragment was purified by gel extraction. For refolding smFRET experiments, a 20 nt biotinylated DNA was annealed to the purified sgRNA by mixing in a 1:1.25 ratio in T50. For vectorial folding smFRET experiments, a 20 nt biotinylated DNA, sgRNA, and a cDNA complementary to a portion of sgRNA were mixed in a 1:1.25:2 ratio in T50. In either case, the sample was heated to 95 °C for 5 min and then cooled to room temperature over 1.5 h. The DNA and RNA sequences are listed in Supporting Information Table S1. Wild-type Cas9 and dCas9 were purchased from IDT.

### Microscopy and Image Acquisition

Total internal reflection microscopy (TIRFM) was performed using a custom prism-based setup. Videos were recorded using EMCCD camera (Andor) and Visual C++ smFRET data acquisition software. Imaging was done at room temperature. Movies were acquired using the red laser excitation at 640 nm for the first and last 10 frames and using the green laser excitation at 532 nm for the remaining frames at 20 frames per second. Short movies were 50 frames long, and long movies were stopped when 80% of molecules have photobleached (∼2000 frames)

### Single-Molecule Data Analysis

**Figure 1:**
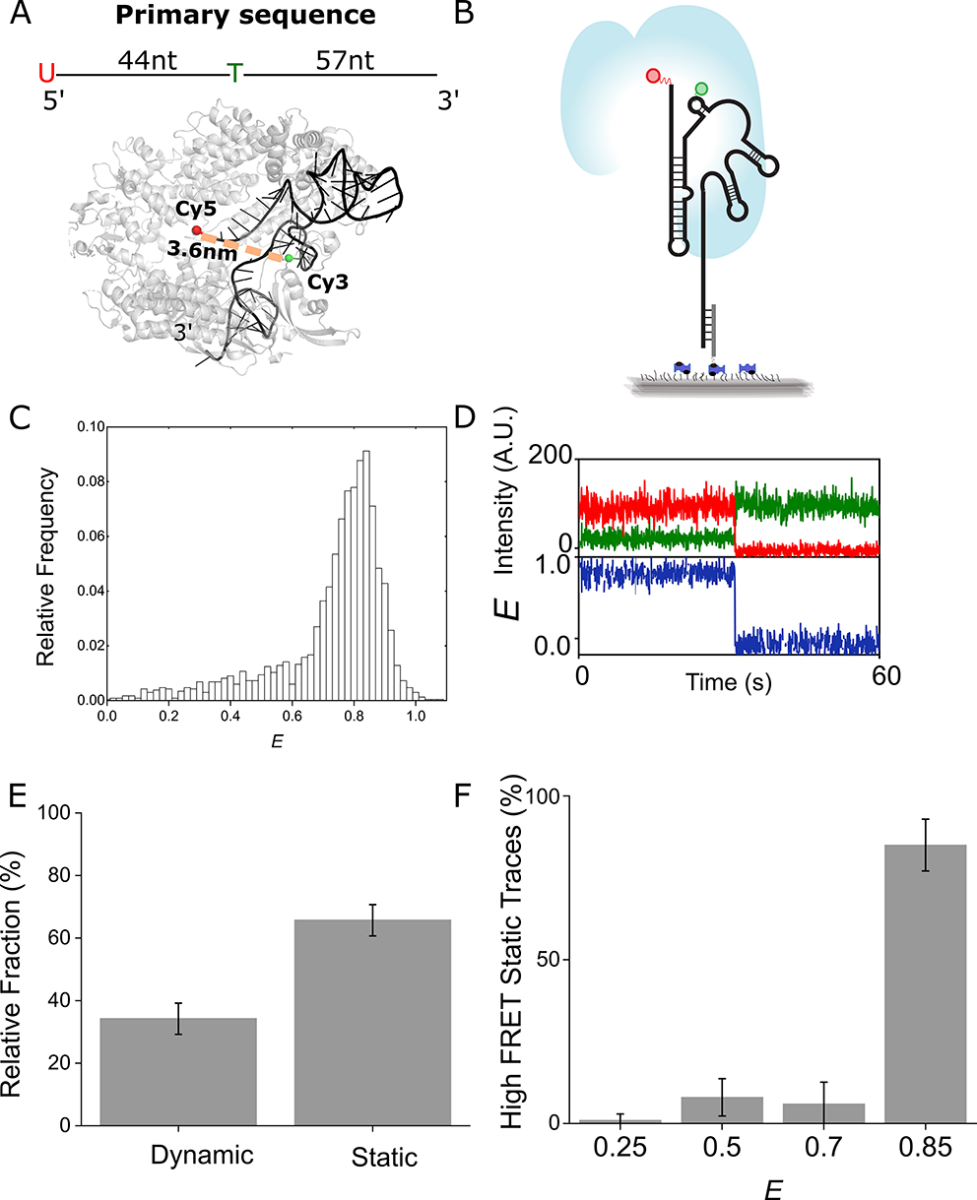
sgRNA dynamics in the context of Cas9 RNP assembly revealed by single-molecule FRET. (A, top) Primary structure of the sgRNA smFRET construct. sgRNA was labeled with Cy3 (green) and Cy5 (red) at the 46th and first nucleotides, respectively. (A, bottom) Crystal structure of spCas9-sgRNA ribonucleoprotein complex organized for target DNA recognition (PDB code 4ZT0). 10nt of the sgRNA’s spacer region was not resolved in the crystal structure. (B) Schematic for smFRET construct to investigate sgRNA dynamics in the context of Cas9 RNP assembly. (C) FRET histograms of Cas9 RNPs with the sgRNA labeled with FRET pairs. There is a major peak at *E* ∼ 0.85. (D) Representative FRET trace sgRNA dynamics when bound by Cas9. Cy5 photobleached at ∼30 s. (E) Comparing relative fraction of static and dynamic trace types. 175 total traces were analyzed by bootstrapping. 66% (±4.6%) of traces were static, and 34% (±4.6%) of traces were dynamic. (F) Comparing relative fraction of static traces by FRET efficiency. 115 total traces were analyzed by bootstrapping. 85% (±7.9%) were *E* ∼ 0.85, 6% (±6.6%) were *E* ∼ 0.7, 8% (±5.7%) were *E* ∼ 0.5, and 1% (±1.9%) were *E* ∼ 0.25.

smFRET movies were analyzed, and traces were extracted using custom software and MATLAB scripts. To select spots, the first 10 frames of direct Cy5 excitation were averaged. The analysis excluded spots that were below and above the empirically determined thresholds. >1000 spots contributed to each histogram. FRET efficiencies were calculated using the *E* = *I*_A_/(*I*_D_ + *I*_A_) where *I*_D_ and *I*_A_ are background subtracted and leakage corrected fluorescence intensities of the donor and the acceptor, respectively. Histograms were analyzed by calculating the normalized fraction folded, which is equal to the relative fraction of molecules within the high FRET region (*E* ∼ 0.8–1.0) divided by the total molecules, excluding those below *E* of 0.4 (*E* ∼ 0.4–1.0). This value was then normalized to the value obtained for Cas9 RNP data from Figure 1 (0.56). The number of molecules used for categorizing traces and folding kinetics is specified in the figures. Dwell times from PIFE to high FRET were manually determined using a custom MATLAB script.

### Single-Molecule FRET Assay

For the RNA refolding assays, sgRNAs were annealed to a DNA tether as mentioned above and diluted to 50 pM in T50. Diluted RNA was immobilized on the polyethylene glycol (PEG)-passivated flow chamber surface (Johns Hopkins University Slide Production Core for Microscopy) using biotin–NeutrAvidin interaction. The chamber was washed with T50 to remove unbound sgRNAs. To capture before-salt images, the chamber was washed with imaging buffer containing 20 mM Tris-HCl, 0.2 mg/mL BSA, 5% [vol/vol] glycerol, 0.8% [wt/vol] dextrose, saturated Trolox [>5 nM],^31^ 1 mg/mL glucose oxidase, and 0.04 mg/mL catalase. To capture molecules in high salt conditions, the chamber was washed with imaging buffer containing the above components plus 100 mM KCl and 5 mM MgCl_2_. For experiments where folding dynamics were captured during addition of salts, imaging continued during exchange of no salt buffer for high salt buffer (in each case containing glucose oxidase and catalase).

For vectorial folding assays sgRNAs were annealed to a DNA tether and cDNA. This was diluted to 50 pM in T50. The diluted sample was immobilized on the PEG-passivated flow chamber surface using biotin–NeutrAvidin interaction. The chamber was washed with T50 to remove free duplexes. The chamber is then washed with a salt free buffer, followed by addition of 50 nM Rep-X incubated for 2 min. To initiate unwinding, we add a buffer containing 20 mM Tris HCl, 0.2 mg/mL BSA, 5% [vol/vol] glycerol, 100 mM KCl, 5 mM Mg^2+^, 1 mM ATP, 0.8% [wt/vol] dextrose, saturated Trolox, 1 mg/mL glucose oxidase, and 0.04 mg/mL catalase (5 nM Cas9 when indicated) to the chamber. To capture folding in real time, we added the buffer during image acquisition.

### Electrophoretic Mobility Shift Assay (EMSA)

An amount of 2 pmol of sgRNA was mixed with 4 pmol of Cas9 and incubated in 10 μL of Cas9 activity buffer at room temperature for 10 min to form Cas9 RNP. An amount of 198 fmol of DNA was added to Cas9 RNP and incubated at 37 C for 30 min. Cas9 RNP-DNA was added to a 2% agarose gel and run for 15 min. The gel was quantified using imageJ.

## Results and Discussion

We studied a sgRNA construct similar to what was used in a crystallographic analysis of Cas9 RNP (Figure 1A).^32^ Both constructs are truncated by 10 nucleotides (nt) in the DNA binding region. This truncation enables Cas9 to bind but not cut DNA. Our construct contains the 3′ most stem loop, which is missing from the sgRNA from crystallography studies. The 5′ most nucleotide of our construct is a uracil rather than the guanine in the previously studied sgRNA. To synthesize the FRET construct, we labeled a 59 nt single-stranded RNA (ssRNA) with Cy3 (donor) and Cy5 (acceptor) and ligated it to a 44 nt ssRNA. We purified the ligated sgRNA using denaturing polyacrylamide to remove undesired products (Figure S1).

The final FRET construct has Cy5 and Cy3 fluorophores at the 1st and 46th positions, respectively. In the crystal structure of Cas9 RNP, the moieties to which the fluorophores are attached to are positioned 3.6 nm apart (Figure 1A).

We validated the activity of our labeled sgRNA using a gel shift assay, where we see similar DNA binding to unlabeled sgRNA (Figure S2).

### sgRNA Dynamics in the Context of an Assembled Cas9 RNP

To examine the sgRNA dynamics in the context of an assembled RNP, we immobilized Cas9 RNPs with our labeled sgRNA construct on a quartz slide by annealing a 20 nt biotinylated DNA tether to a complementary 3′ extension on the sgRNA (Figure 1B). Previous smFRET studies showed that this immobilization strategy does not disrupt Cas9 RNP activity.^33−35^ To uncover the distribution of sgRNA conformations, we generated a histogram by binning the average *E* values of thousands of molecules over a 0.5 s (10 frames) window. The histogram shows a single major peak at *E* ∼ 0.85, suggesting that most molecules fold into a compact structure (Figure 1C). This high FRET value is consistent with our expectations given the 3.6 nm distance plus the dye linkers. To examine if sgRNAs remained in one conformation or were dynamic, we analyzed 175 time trajectories each 60 s long (Figure 1D, Figure S3**)**. We categorized them as either static or dynamic. Dynamic traces had at least one FRET transition during the 60 s. Due to practical reasons such as fluorophore photobleaching, we cannot observe single molecules indefinitely. We therefore used 60 s, our typical duration of data acquisition in a single field of view, as the time scale against which our classification is made. 66% (±4.6%) of molecules were static, while 34% (±4.6%) of molecules were dynamic (Figure 1E), suggesting that sgRNAs may still be flexible when bound to Cas9.^36^ To determine what FRET states static sgRNAs were steady in, we analyzed 115 steady FRET time trajectories. Most traces, 85% (±7.9%), were steady high FRET at *E* ∼ 0.85. However, a small fraction showed static FRET in other states; 6% (±6.6%) were steady *E* ∼ 0.7, 8% (±5.7%) *E* ∼ 0.5, and 1% (±1.9%) *E* ∼ 0.25 (Figure 1F). These other states are consistent with sgRNAs being in conformations that cause the FRET pair to be further apart and may represent sgRNA conformations that are suppressed by Cas9 binding.

### sgRNA Dynamics after Salt-Induced Refolding

**Figure 2:**
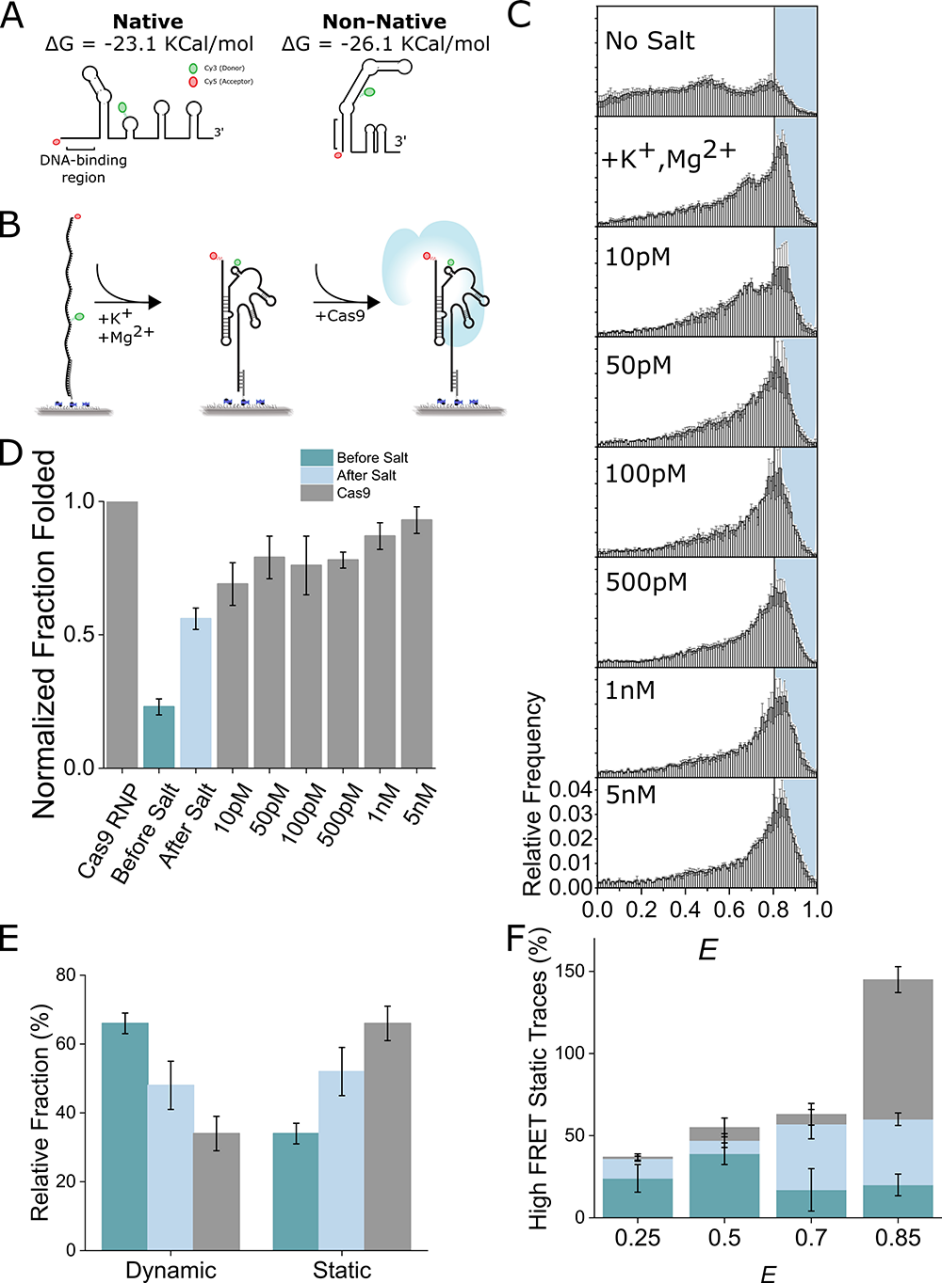
Cas9 increases relative high FRET to mid-high FRET fraction. (A) mFold predictions of sgRNA secondary structures. The native secondary structure (Δ*G* = −23.1 kcal/mol) is output only when the DNA binding regions of sgRNAs are restricted to being single stranded. Non-native secondary structures are output when no restrictions are input. The non-native structure that is more stable than the native structure (Δ*G* = −26.1 kcal/mol) is pictured. (B) Schematic of smFRET assay to measure sgRNA dynamics without salts, after K^+^/Mg^2+^ addition, and after Cas9 binding. (C) FRET histograms as a function of condition. Shaded fraction highlighted from *E* ∼ 0.8 to 1.0. (D) Fraction folded as a function of salt and Cas9 concentration. Folded fraction [(*E* > 0.8)/(0.4 > *E* > 1.0)] of sgRNAs compacted into a high FRET structure. Values were as follows: before salt,: 0.13 ± 0.01; after salt, 0.32 ± 0.02; 10 pM Cas9, 0.4 ± 0.05; 50 pM Cas9, 0.45 ± 0.04; 100 pM Cas9, 0.43 ± 0.06; 500 pM Cas9, 0.44 ± 0.02; 1 nM Cas9, 0.49 ± 0.03; 5 nM Cas9, 0.53 ± 0.03. (E) Comparing relative fraction of static and dynamic trace types per condition. 290 total traces were analyzed by bootstrapping. No salt: 34% (±3%) of traces were static and 66% (±3%) of traces were dynamic. After salt: 52% (±6.6%) of traces were static and 48% (±6.6%) of traces were dynamic. With Cas9: 66% (±4.6%) of traces were static and 34% (±4.6%) of traces were dynamic. (F) Comparing relative fraction of static traces by FRET efficiency per condition. 132 total traces were analyzed by bootstrapping. No salt: 20% (±8.4%) were *E* ∼ 0.85, 17% (±6.6%) were *E* ∼ 0.7, 39% (±12.9%) were *E* ∼ 0.5, and 24.2% (±6.6%) were *E* ∼ 0.25. After salt: 40% (±3.8%) were *E* ∼ 0.85, 40% (±8.9%) were *E* ∼ 0.7, 8% (±4.2%) were *E* ∼ 0.5, and 12% (±1.6%) were *E* ∼ 0.25. With Cas9: 85% (±7.9%) were *E* ∼ 0.85, 6% (±6.6%) were *E* ∼ 0.7, 8% (±5.7%) were *E* ∼ 0.5, and 1% (±1.9%) were *E* ∼ 0.25. Error bars are standard error of biological replicates.

The sgRNA sequence tested is predicted to fold into a non-native secondary structure more stably (Δ*G* = −26.1 kcal/mol) than the native secondary structure (Δ*G* = −23.1 kcal/mol) (Figure 2A, Figure S4).^36^ Therefore, we expect that some of the sgRNAs will adopt non-native tertiary conformations. In principle, these sgRNAs may have the same FRET values as native conformations. However, our observation of low abundance static traces with lower values (*E* ∼ 0.7, 0.5, and 0.25) suggests that Cas9 binding may suppress these FRET states arising from other sgRNA conformations. To determine if these lower FRET static traces are more abundant in the absence of Cas9, we immobilized sgRNAs without Cas9 (Figure 2B). Under no salt conditions, histograms show a broad distribution of FRET states (Figure 2C). This is not surprising because monovalent and divalent cations are required for stabilizing sgRNA structures.

We wondered if adding salts would be sufficient to stabilize sgRNAs into a single high FRET population at *E* ∼ 0.85. After adding 100 mM KCl and 5 mM MgCl_2_ to the sgRNAs, the distribution shifted toward higher FRET values and narrowed. However, there are two high FRET peaks rather than one seen with Cas9 bound sgRNAs: one high FRET, major peak at *E* ∼ 0.85 (dark gray) and a minor peak at *E* ∼ 0.7 (Figure 2C). The minor peak represents sgRNAs that are in a conformation that increases the distance between the fluorophores. To quantify this shift, we calculated fraction folded, which is equal to molecules greater than (or equal to) *E* ∼ 0.8 divided by molecules greater than (or equal to) *E* ∼ 0.4 (Figure 2C). To compare histograms across conditions through quantification of the native-like fractions, we chose to use a single threshold, *E* of 0.8, because, although this may not accurately estimate the absolute fraction of each population, we can use the same threshold across different experiments and experimental conditions so that our relative changes are reported reliably. For sgRNA with salt, the fraction folded was 0.56 ± 0.04 (Figure 2D).

To determine if sgRNAs under these two conditions were mostly static in a range of conformations or sampling multiple conformations, we analyzed 209 time trajectories before and after adding salt. After adding salts, the static fraction increased by 53%, reflecting the ion-dependent stabilization of RNA structures (Figure 2E). To examine how the distribution of static FRET conformations changed after adding K^+^ and Mg^2+^ ions, we analyzed 132 static trace time trajectories. In these conditions the *E* ∼ 0.25 and 0.5 static fractions decreased by 50% and 79%, respectively (Figure 2F). Conversely, the *E* ∼ 0.7 and 0.85 static fractions increased by 135% and 100%, respectively (Figure 2F). This suggests that ions promote sgRNAs stabilization into high FRET compact conformations. However, 48% of traces in high salt were dynamic, indicating that sgRNA can remain dynamic without Cas9.

### Cas9-Dependent Changes to sgRNA Dynamics

To measure the Cas9 dependent changes to sgRNA dynamics, we immobilized sgRNAs and folded them by addition of ions. Then we added Cas9 in increasing concentrations from 10 pM to 5 nM. We observe a concentration dependent increase in the normalized fraction folded. This value increased by 66% from 0 nM to 5 nM (Figure 2C,D). Static traces were also 65% more abundant for sgRNAs with Cas9 compared to no Cas9 (Figure 2E). Static traces at *E* ∼ 0.85 also increased in abundance by 113% compared to no Cas9 (Figure 2F). These results demonstrate that Cas9 can suppress dynamic sgRNAs and promotes a compact sgRNA conformation.

### Vectorial Folded sgRNA Dynamics

**Figure 3:**
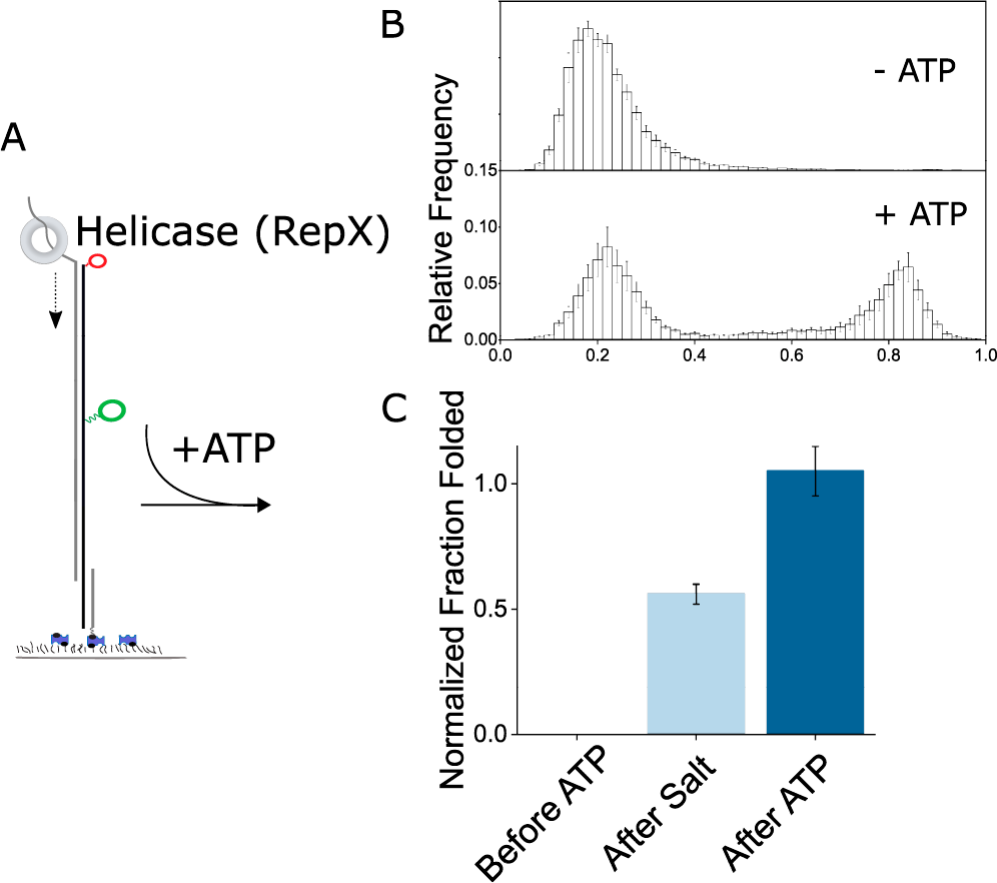
Vectorial folded sgRNA FRET distribution is different than refolded sgRNAs. (A) Schematic of vectorial folding assay for co-transcriptional folding of sgRNAs. Shown is a sgRNAs and complementary DNA construct with an ATP-dependent helicase (Rep-X). Addition of ATP activates Rep-X, mediating removal of cDNA and co-transcriptional folding of sgRNA. (B) FRET histograms of vectorial folded sgRNA labeled with FRET pairs. There is a major peak at *E* ∼ 0.85. (C) Folded fraction (dark gray) across conditions comparing refolding to vectorial folding. Values were as follows: before ATP, 0.0 ± 0.0; after salt, 0.56 ± 0.04; after ATP, 1.05 ± 0.05.

Unlike a chemically synthesized RNA, sgRNAs transcribed from a DNA template can start to fold during transcription. This co-transcriptional folding is an example of vectorial folding. To examine if vectorial-folded sgRNA’s dynamics were the same as presynthesized sgRNAs, we used a previously developed helicase-based vectorial RNA folding assay to fold the sgRNA.^30,37^ Here we annealed a sgRNA molecule to a complementary DNA (cDNA) strand and immobilized the duplex via a biotin tether. The cDNA strand contained a 13 nt poly thymine track at the 3′ end for loading a Rep-X superhelicase^37^ (Figure 3A). As expected, before adding ATP, there was a single peak at *E* ∼ 0.2 representing the sgRNA:cDNA duplexes (Figure 3B). After adding ATP, we observed the emergence of a second peak at *E* ∼ 0.85, representing the sgRNA in a compact conformation after cDNA removal. Strikingly, we did not observe a peak at 0.7 as in previous experiments with the presynthesized sgRNA, despite being in similar salt conditions (Figure 3B). The fraction folded was 88% higher for vectorial folding than for refolding by salts (Figure 3C). The peak at 0.2 remains because some RNA/DNA duplexes were not unwound by Rep-X. We only observed the emergence of the *E* = 0.85 peak after adding a buffer containing both Rep-X and ATP, confirming that the sgRNA folding was dependent on Rep-X unwinding the duplex (Figure S5).

### Cas9 Impact on sgRNA Folding Kinetics

**Figure 4:**
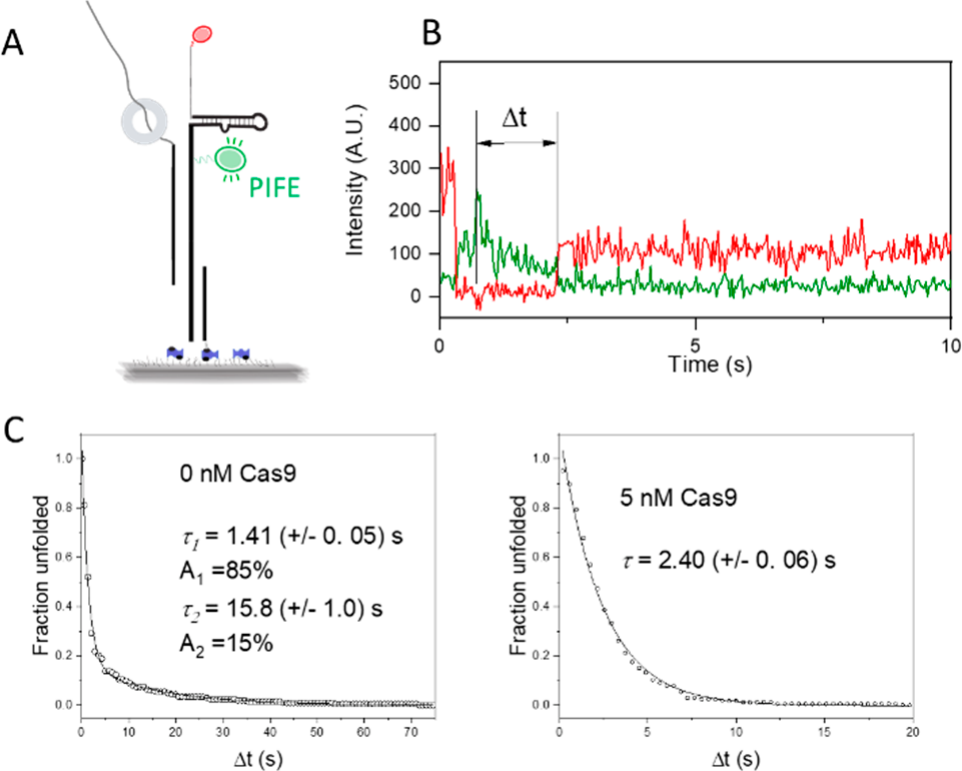
Cas9 enhances sgRNA folding kinetics. Cas9 removes slow folding sgRNA fraction. (A) Schematic of real-time vectorial folding assay for measuring sgRNA folding kinetics. Cartoon captures moment of Rep-X passing by Cy3 fluorophore causing protein induced fluorescent enhancement. (B) Representative trace of real time sgRNA folding. Time difference Δ*t* between PIFE event and sgRNA folding event is marked. Initial spike in Cy5 is caused by its direct excitation to confirm Cy5 presence. (C) Survival histograms showing the folding kinetics of sgRNAs in the presence and absence of 5 nM Cas9. A double exponential decay was used to fit the no Cas9 curve with the lifetimes and relative populations indicated, whereas a single exponential decay was sufficient to fit the curve with Cas9.

To measure sgRNA folding kinetics, we used protein induced fluorescent enhancement (PIFE)^38,39^ as a marker for Rep-X traversing past Cy3 on nucleotide 46 (Figure 4A). When we looked at sgRNA vectorial folding in real time, we observed this PIFE effect, and most sgRNA molecules subsequently folded in a single step (Figure 4B, Figure S6). We measured the time from PIFE to stable high FRET of 202 molecules, whose distribution could be best fit using a double exponential decay function with the slow lifetime component with the lifetime τ_2_ of 15.8 s representing 15% of molecules that showed folding (Figure 4B,C). We wondered if Cas9 had any impact on folding kinetics. To test this, we added Cas9, in saturating concentration of 5 nM, during sgRNA folding. Cas9 eliminated the slow folding fraction, and the cumulative histogram of the time spent in the unfolded state after PIFE could be fit well using a single exponential decay with the lifetime τ of 2.4 s (Figure 4C).

## Conclusions

In this study, we used smFRET to study the sgRNA folding dynamics in the context of CRISPR Cas9 RNP assembly. This approach allowed us to measure the heterogeneity in the population of sgRNAs under various conditions. We also recorded individual sgRNA folding dynamics in real time. Notably sgRNAs were the most dynamic in a no salt buffer and had the broadest range of FRET efficiencies. This can be rationalized by the multiple predicted secondary structures for our sgRNA sequence (Figure 2A, Figure S4). Each secondary structure may lead to a different tertiary structure. Presynthesized sgRNA can form a diverse set of interactions, including long-range base pairing between the 5′ and 3′ regions. Under low ionic conditions, these interactions are not stable allowing sgRNAs to remain flexible.

We detected two major FRET populations under high salt conditions, suggesting that at least one other tertiary structure can form with the sgRNA. We also detected both dynamic and static sgRNAs under these conditions, confirming that the sgRNA remains flexible even in conditions that can stabilize RNA structures. The DNA binding region pairing with the sgRNA scaffold has been shown to drive sgRNA misfolding.^10^ The DNA binding region of our sgRNA (nucleotides 2–4 and 6–10) is predicted to partially pair with nucleotides 44–53 of the backbone. This base pairing can potentially prevent the first stem loop from forming in a refolding experiment (Figure S4). However, all other stem loop structures are predicted to form. Perhaps Cas9 still binds and compacts these RNAs into high FRET structures. However, these RNPs could be inactive.

Previous work has also shown that RNAs can have different folding outcomes depending on how they are folded.^29,22,40^ We observed a single FRET population for vectorial folded sgRNAs contrary to refolded sgRNAs. This is consistent with previous studies that show differences in cleavage activity of Cas9 RNA if the same sgRNAs is refolded versus vectorial folded, i.e., in vitro transcribed RNA is used as is.^10^ More than half of the sgRNAs they tested had improved cleavage activity after refolding. For example, one sequence showed a 7-fold improvement in cleavage activity after refolding. However, effects were sequence specific. Notably, one sgRNA’s activity was abolished after refolding. Our work directly shows a difference in sgRNA folding depending on how it is folded, which may explain their observation of differences in Cas9 cleavage activity. One way to improve the activity of sgRNAs can be to heat sgRNAs to refold them into an active structure, assemble them with Cas9, and deliver RNPs rather than nucleic acids.^10^ However, this is only useful if the native structure is more thermodynamically favorable than the non-native structure. Collectively, differences in activity attributable to sgRNA folding could arise when sgRNAs are delivered as a DNA plasmid, as a naked RNA, or in an RNP complex. Our results are consistent with previous work that suggested that sgRNA folding is variable even within one sequence and could contribute to variability in genome editing outcomes even for one sgRNA-DNA target pair.^10,41,42^ The approaches used include base substitutions in the RNA backbone to break noncanonical interactions with the DNA binding region, extending the duplex between the crRNA and tracrRNA to improve Cas9 binding and introducing a stable hairpin to make folding more robust. Furthermore, Cas9 enzymes from other species with their own distinct gRNAs have been discovered.^43,44^ Our assay could be extended to study other gRNAs and inform rational design of future engineered variants.

## Supporting Information Available

Figure S1 showing ligation scheme and PAGE gel for smFRET construct; Figure S2 showing EMSA comparing DNA binding between labeled and unlabeled sgRNA; Figure S3 showing example FRET time trajectories; Figure S4 showing predicted secondary structures of sgRNA from Mfold; Figure S5 showing vectorial folding control histograms; Figure S6 showing Kymograph heatmap of vectorial folding in real-time; Table S1 listing DNA and RNA sequences used in this study (PDF)
