# Supplementary material for: Single Molecule FRET Analysis of CRISPR Cas9 Single Guide RNA Folding Dynamics
Source: J Phys Chem B. 2022 Dec 23;127(1):45–51. doi: 10.1021/acs.jpcb.2c05428 (PMC9841515; doi:10.1021/acs.jpcb.2c05428)
Supplement: Supplementary file 1 — jp2c05428_si_001.pdf [file jp2c05428_si_001.pdf]

Supplementary Information for

Single Molecule FRET Analysis of CRISPR Cas9 Single Guide RNA Folding Dynamics

Ikenna C. Okafor[1] and Taekjip Ha[2-5, @]

[1]Department of Biology, Johns Hopkins University, Baltimore, Maryland 21218, USA

[2]Department of Biophysics and Biophysical Chemistry, Johns Hopkins University School of Medicine, Baltimore, Maryland 21205, USA.

[3]Department of Biophysics, Johns Hopkins University, Baltimore, MD 21218, USA.

[4]Department of Biomedical Engineering, Johns Hopkins University, Baltimore, Maryland 21205, USA.

[5]Howard Hughes Medical Institute, Baltimore, Maryland 21205, USA.

[@]To whom correspondence should be addressed: Taekjip Ha (tjha@jhu.edu)

Table S1

RNA Substrates	Sequence (5' -> 3')
sgRNA fragment #1	UAUGAGACGCGUUUUAGAGCUAGAAAUAGCAAGUUAAAAUAAGGCTAGUCCGUUAUCA
sgRNA fragment #2	CUUGAAAAAGUGGCACCGAGUCGGUGCUUUUUUUGCUCGUGCGC
Ligated sgRNA	UAUGAGACGCGUUUUAGAGCUAGAAAUAGCAAGUUAAAAUAAGGCTAGUCCGUUAUCA<br>CUUGAAAAAGUGGCACCGAGUCGGUGCUUUUUUUGCUCGUGCGC
**DNA Substrates**	
DNA Splint for sgRNA ligation<br>5'-<br>GCGCACGAGCAAAAAAAGCACCGACTCGGTGCCACTTTTTCTAACGGACTAGAATTATTTTAACTTGCTATTTCT<br>AGCTCTAAAACGCGTCTC -3'	
20-nt Biotin ssDNA tether<br>5' - /5Biosg/AAGATCCAGCGCACGAGCAA – 3'	
cDNA for vectorial folding assay<br>5' –<br>AAGCACCTCGGTGCCACTTTTTCAAGTGATAACGGACTAGCCTTATTTTAACTTGCTATTTCTAGCTCTAAAACGC<br>TCATATTTTTTTTTTTTTT - 3'	
dsDNA for EMSA<br><br>5' – GCACAGCAGAAATCTCTGCTGACGCATAAATATGAGACGCTGGAGTACAAACGTCAGCTTGCT -3'<br>3' – CGTGTCGTCTTTAGAGACGACTGCGTATTAATACTCTGCGACCTCATGTTTGCAGTCGAACGA -5'	

RNA and DNA substrates used in this study U represents a uracil with Cy5 attached to the 5' phosphate. T represents modified thymine /iAmMC6T/ with Cy3 labeling. /5Biosg/ represents a 5' biotin attachment. G represents a guanine with Cy3 attached to the 5' phosphate. GGA represents the PAM sequence on the DNA substrate.

Figure S1

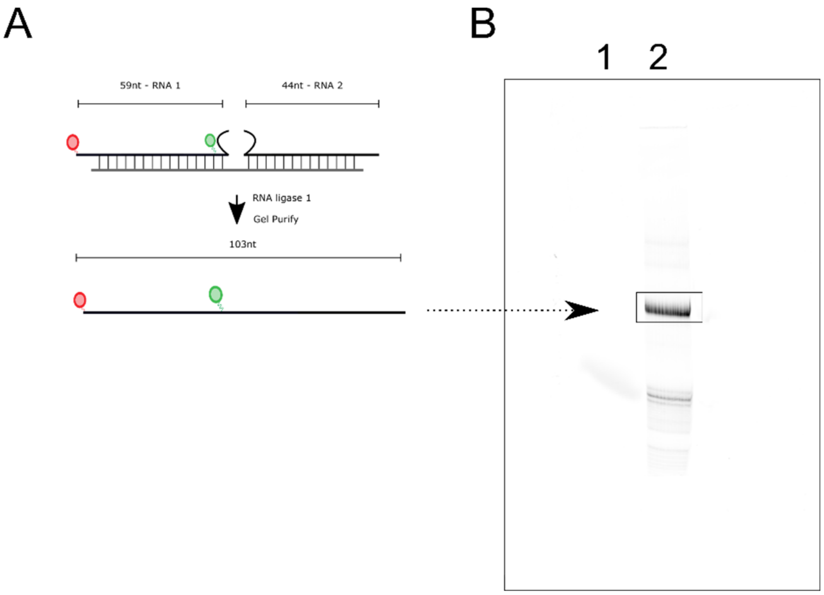

PAGE Gel purification of FRET labeled sgRNA. (A) schematic of ligation of two ssRNA fragments with Cy3 and Cy5 labeling. ssRNA was annealed to a DNA splint creating ssRNA overhangs for RNA ligase 1 to use as a substrate. The final product is a 103nt ssRNA. (B) 12% PAGE denaturing gel. Lane 1 is 1X RNA loading dye with Xylene Cyanol running at ~55bp. Lane 2 with 103nt fragment boxed and 59nt below.

Figure S2

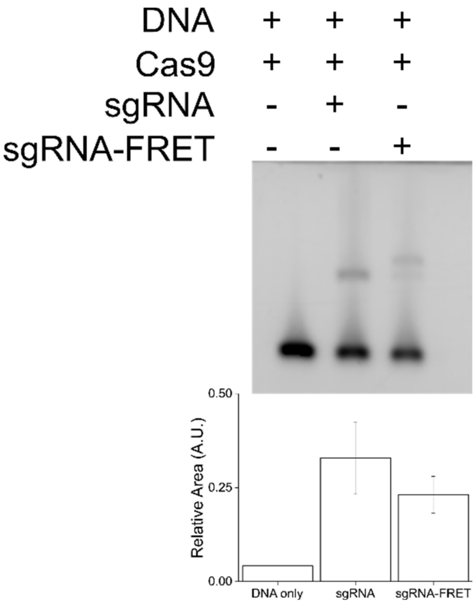

smFRET labeled sgRNA enables Cas9 dependent DNA binding. (A) 2% agarose gel (top). Lane 1 DNA and Cas9, Lane 2 DNA and Cas9 RNP (unlabeled sgRNA), Lane 3 DNA and Cas9 RNP (labeled sgRNA). Intensity of each lane was measured. Relative area is equal to the intensity of the shifted band relative total intensity of the shifted and unshifted bands. Relative area in decreasing order was Cas9-sgRNA 0.33 ± 0.01 (lane 2), Cas9 sgRNA-FRET 0.23 ± .05 (lane 3) and Cas9 only 0.04 ± 0.0 (lane 1).

Figure S3

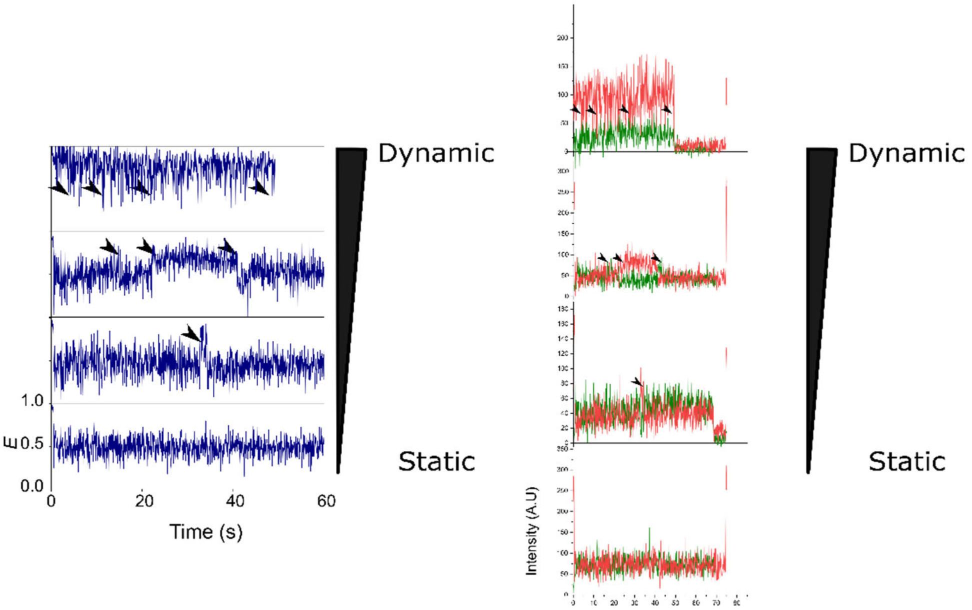

A B

Example traces showing diverse behaviors. (A) Time trajectories showing 60 seconds of FRET and Cy3 and Cy5 fluorescence for molecules showing the range of behaviors from dynamic (top) to static (bottom). Traces fluctuate between values *E* 0.85 to *E* 0.25 or is static in values between this range. Arrows designate FRET transitions on both the FRET and intensity plots.

Figure S4

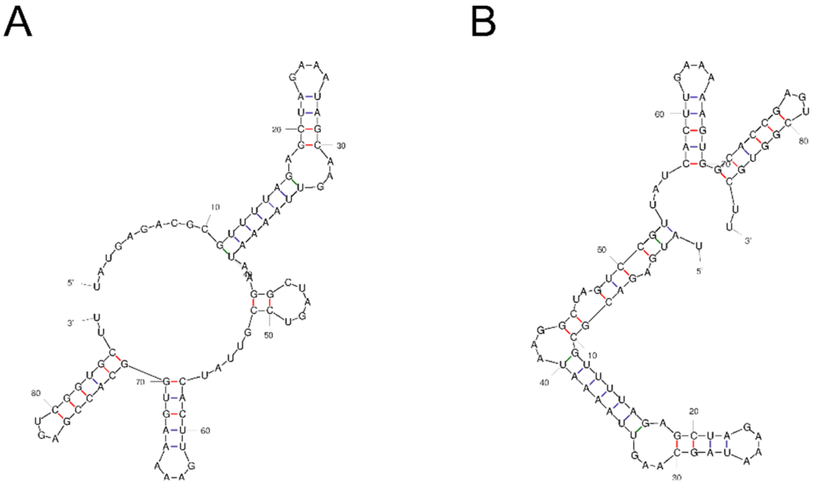

Mfold predictions of sgRNA. (A) Native structure predicted by Mfold (ΔG = -23.1 kcal/mol). Nucleotides 1 – 10 (DNA binding region) were constrained to being single stranded. Folding temperature was 37°C, salt conditions were 1M NaCl. Maximum bulge loops were set to 30, there was no limit to maximum distance between paired bases. Percent suboptimality was 5 and upper bound on the number of computed foldings was set to 50. (B) Nonnative structure predicted by Mfold (ΔG = -26.1 kcal/mol). Parameters were the same as above besides the constraint on nucleotides 1 -10.

Figure S5

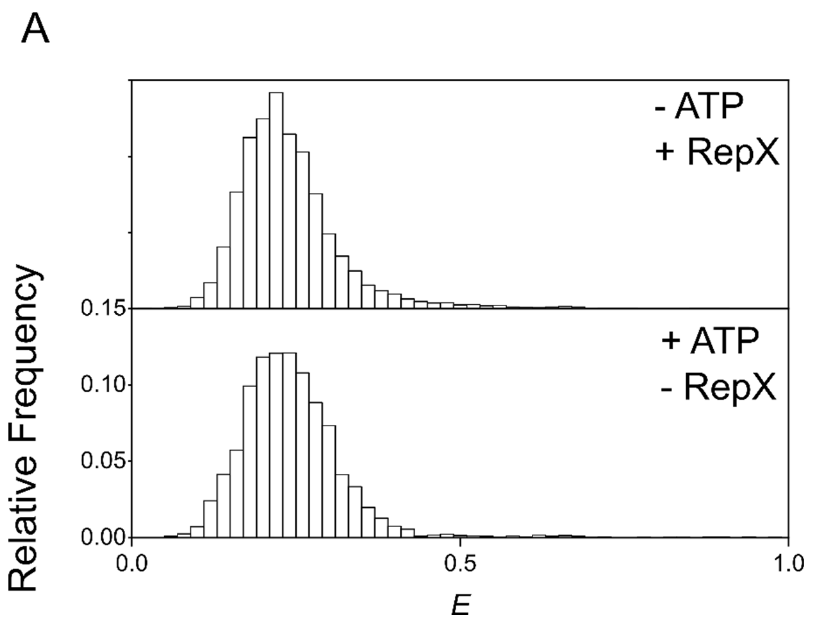

Vectorial folding requires ATP and Rep-X. (A) FRET histogram showing the distribution of efficiencies after adding unwinding buffer with no ATP (top) or without adding Rep-X prior (bottom). The peak at *E* ~0.2 is the cDNA:sgRNA duplex.

Figure S6

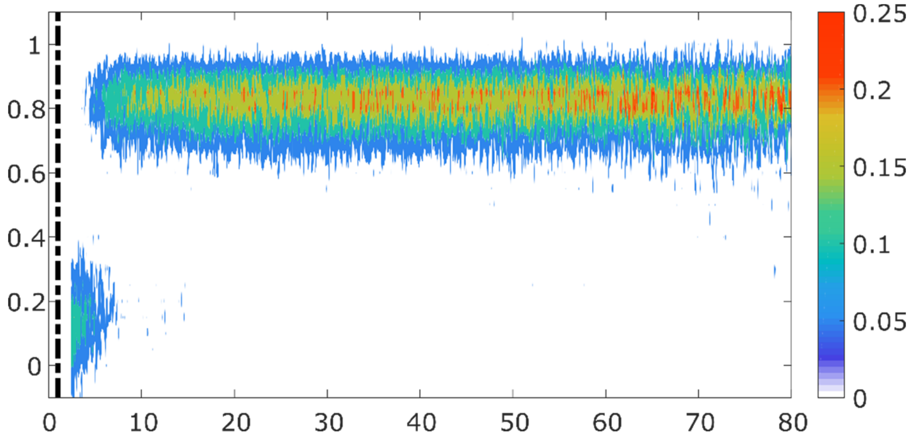

Cumulative trace of vectorial folded sgRNA. Overlay of 133 FRET time trajectories aligned by the center of the protein induced fluorescent enhancement (PIFE) peaks. Dotted indicates addition of ATP containing unwinding buffer.
